# SARS-CoV-2 vaccine excipients polyethylene glycol and trometamol do not induce mast cell degranulation, in an *in vitro* model for non-IgE-mediated hypersensitivity

**DOI:** 10.3389/falgy.2022.1046545

**Published:** 2022-12-20

**Authors:** Paola Leonor Quan, Laia Ollé, Marina Sabaté-Brescó, Yanru Guo, Rosa Muñoz-Cano, Annette Wagner, Gabriel Gastaminza, Margarita Martín

**Affiliations:** ^1^ Department of Allergy and Clinical Immunology, Clínica Universidad de Navarra, Pamplona, Spain; ^2^ Laboratory of Clinical and Experimental Respiratory Immunoallergy, IDIBAPS, Barcelona, Spain; ^3^ Biochemistry Unit, Biomedicine Department, Faculty of Medicine, University of Barcelona, Barcelona, Spain; ^4^ Research Network on Asthma, Drug Adverse Reactions and Allergy (Red de Investigación en Asma, Reacciones Adversas a Fármacos y Alergia) (ARADyAL), Madrid, Spain; ^5^ Instituto de Investigación Sanitaria de Navarra (IdiSNA), Pamplona, Spain; ^6^ Allergy Department, Hospital Clinic, University of Barcelona, Barcelona, Spain; ^7^ Department of Adult Allergy, Guy’s and St Thomas’ NHS Foundation Trust, London, United Kingdom

**Keywords:** SARS-CoV-2 vaccines, polyethylene glycol, trometamol, mast cells, non-IgE-mediated hypersensitivity mast cell activation, MRGPRX2

## Abstract

The development of vaccines against SARS-CoV2 brought about several challenges, including the management of hypersensitivity reactions to these formulations. The search for underlying mechanisms involved in these adverse events initially focused on excipients which may trigger mast cell activation responses via non-IgE pathways: polyethylene glycol and trometamol. We sought to determine whether these components, in their pure form, were capable of stimulating mast cells directly. To test this hypothesis, we used an in vitro model for non-IgE-mediated activation that has previously shown degranulation responses induced via MRGPRX2 with known drug agonists of the receptor. Human LAD2 mast cells were incubated with different concentrations (1, 10, 50 mg/ml) of trometamol and of purified polyethylene glycol/Macrogol (molecular weights: 2,000, 3,350, 4,000, and 6,000). Mast cell degranulation was assessed using a beta-hexosaminidase read-out. Interestingly, degranulation responses for all reagents tested showed no significant differences from those obtained from the negative control (basal degranulation). Receptor-silencing assays were therefore not conducted. In summary, purified PEG and trometamol did not induce mast cell degranulation in this in vitro model for the study of non-IgE mechanisms of drug hypersensitivity, previously shown to be useful in the investigation of MRGPRX2 ligands. Studies using complete vaccine formulations, lipid conjugates, and receptor gene variants are needed to further clarify mechanisms of vaccine hypersensitivity.

## Introduction

The fast-paced development of vaccines against SARS-CoV2 brought about new challenges for clinicians and allergists, including the occurrence of hypersensitivity reactions to these formulations. Regarding immediate-type reactions, symptoms ranging from mild to severe were reported, and the search for underlying pathogenic mechanisms is ongoing. Initially, focus turned towards excipients present in SARS-CoV2 vaccines that are capable of triggering hypersensitivity (1–3). Two compounds have received most of the attention: polyethylene glycol (PEG), a polymer compound found in the Pfizer-BioNTech and Moderna COVID-19 vaccines, and tris (hydroxymethyl)aminomethane (also known as trometamol, or tromethamine), an organic amine found in the Moderna vaccine. Polysorbate 80, structurally related to PEG with potential cross-reactivity (4), is found in both the Astra-Zeneca and Janssen COVID-19 vaccines.

PEG, PEGylated products, and polysorbates, which share similar structures, have been identified as inducers of hypersensitivity reactions, mostly of immediate-type (4, 5), with clinical characteristics compatible to those produced by SARS-CoV2 vaccines. The involved mechanism of hypersensitivity is only partially understood. Recent studies showing cross-reactivity between different pegylated compounds (4, 5), and the identification of IgE against PEG in the context of anaphylaxis (6), as well as evidence of antigen-specific inhibition of histamine release by monomeric and dimeric fractions of PEG (7), suggest that an immunoglobulin E (IgE)-mediated pathway is involved. On the other hand, variable results have been observed in terms of skin testing positivity to PEG and PEG-related products (4), and adverse reactions often occur upon first documented exposure to a specific PEG-containing formulation. The other compound, trometamol, has been described as a causative agent of anaphylaxis in gadolinium-based formulations (8). Positive skin-testing with trometamol has recently been described in patients with delayed local reactions to the Moderna mRNA SARS-CoV2 vaccine (9). Interestingly, these patients experienced earlier-onset, but milder, local reactions after their second vaccine dose (9). Larger studies regarding hypersensitivity mechanisms for trometamol are missing. Finally, a publication by Krantz et al., reports tolerance to a second dose of a SARS-CoV2 vaccine in a cohort of 189 patients who experienced immediate reactions to a first dose of the vaccine (10). Given the evidence described above, we believe that an exploration of non-IgE mechanisms for vaccine hypersensitivity is justified.

Recently, our group has shown that several drugs are capable of inducing mast cell (MC) degranulation *in vitro* directly, as measured by beta-hexosaminidase release (11). Furthermore, receptor silencing experiments showed that many of these compounds induce such activation via the Mas-related G protein-coupled receptor member X2 (MRGPRX2), a receptor found mainly in connective tissue MCs and identified as a cause for pseudo-allergic, immediate-type reactions to drugs, as well as other immune and allergic conditions (11). MRGPRX2 is also a known target for specific drug groups with common structural elements (11). The newly demonstrated relevance of this receptor in drug hypersensitivity highlights the importance of investigating non-IgE pathways for MC activation by drugs and excipients. We therefore considered it important to investigate whether PEG and trometamol are capable of stimulating MCs directly. To meet this objective, we used a model for non-IgE-mediated activation that has previously that has previously revealed degranulation responses *via* MRGPRX2 (11). The model employed requires two steps. First, to observe whether compounds activate MCs directly, in a way not specific to any receptor, by characterizing the degree of degranulation induced in human MCs (LAD2 cell line), by the presence of drugs in incubation. Second, if degranulation is found, to demonstrate the involvement of a specific receptor pathway by confirming its presence in the cell and then silencing it. In the case of MRGPRX2, this involves a lentiviral knock-down system and confirmation of receptor silencing using Western Blot and flow cytometry (11). The second step depends on finding activation in the first, which we completed using the method explained below.

## Methods and results

**Figure 1:**
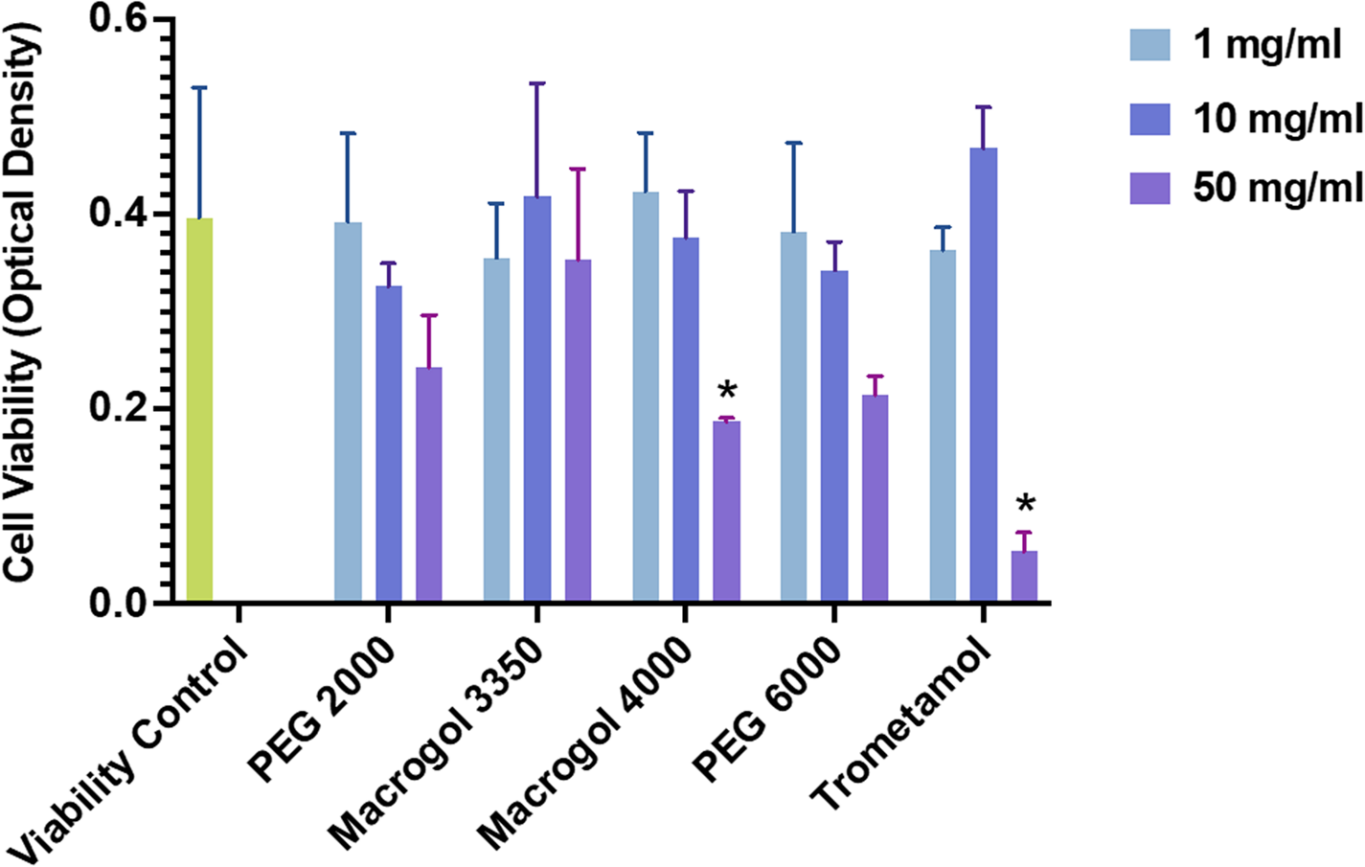
Viability of LAD2 MCs after 1 h of incubation with polyethylene glycol/macrogol (molecular weights of 2,000, 3,350, 4,000, 6,000) and trometamol at different concentrations (1, 10, 50 mg/ml), expressed in absorbance units (as measured using the WST-1 assay protocol). Statistically significant differences (*p*<0.05) are marked with asterisks.

Human LAD2 MCs were incubated with different concentrations (1, 10, 50 mg/ml) of trometamol and of PEG/Macrogol (molecular weights: 2,000, 3,350, 4,000, and 6,000), to evaluate cell viability (Figure 1). The LAD2 human MC line kindly provided by Drs. A. Kirshenbaum and D.D. Metcalfe (National Institutes of Health, Bethesda, MD) was grown in StemPro-34 media (Life Technologies, Carlsbad, CA), supplemented with StemPro-34 nutrient and L-glutamine (2mM), penicillin (100 U/mL) and streptomycin (100 μg/mL), and 100 ng/mL SCF (Amgen, Thousand Oaks, CA) (12). Viability was studied using the WST-1 protocol, which consists of a colorimetric assay that measures the absorbance of formazan dye (expressed in units of optical density) produced by cellular mitochondrial dehydrogenases in viable cells as they convert the tetrazolium salt WST-1. Unstimulated cells cultured in cell medium (Stem-Pro) were used as negative controls. We seeded 5 × 10^4^ cells for each point in a 96-well plate (Greiner Bio-One) and incubated for 1 h at 37°C with different concentrations of the specified reagents. Then, 10% WST-1 reagent (Roche Diagnostics, GmbH-Germany) was added to each well and the plate was incubated for a further 2 h at 37°C. Absorbance was read at 450 nm. Cell viability was conserved for all reagents at concentrations of 1 and 10 mg/ml, and significantly reduced at concentrations of 50 mg/ml for all reagents, except for Macrogol 3350.

**Figure 2:**
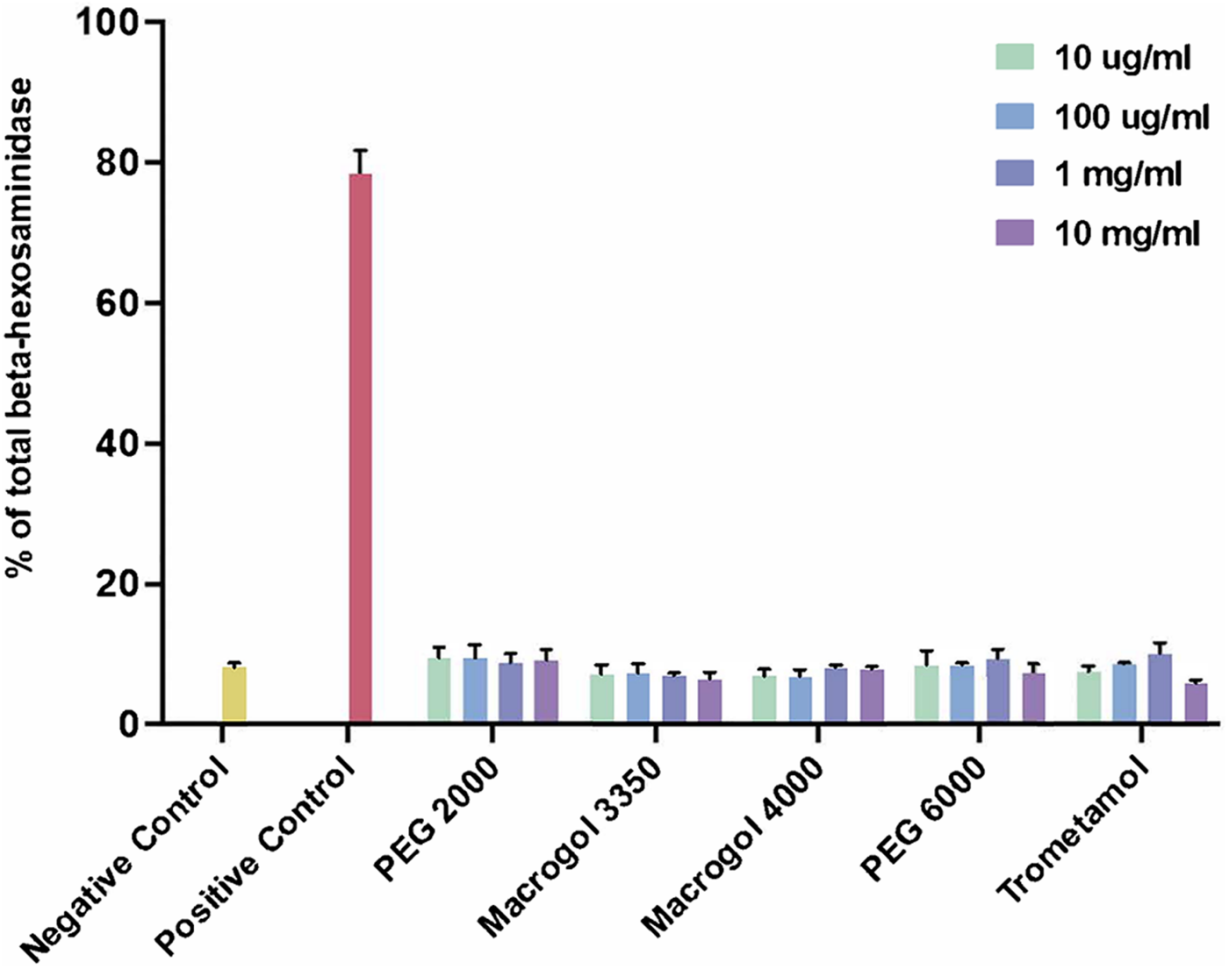
Percentage of total beta-hexosaminidase release after incubation with reagents (polyethylene glycol/macrogol and trometamol) at different concentrations. Unstimulated cells were used as a negative control (basal degranulation), cells stimulated with phorbol 12-myristate 13-acetate (PMA) (10 ng/ml) and ionomycin (0.5 µM) were used as a positive control.

We then proceeded to test the ability of the compounds to induce MC degranulation at several concentrations (Figure 2). We employed 10 mg/ml as the highest concentration, guided by our cell viability results. The procedure was completed as follows: A total of 3 × 10^4^ cells per well were incubated in a 96-well plate (Greiner Bio-One) during 30 min, at 37°C, with Tyrode's buffer (negative control, indicative of basal degranulation), with different concentrations (10 µg/ml, 100 µg/ml, 1 mg/ml, 10 mg/ml) of the reagents (PEG, Macrogol, trometamol), and with phorbol 12-myristate 13-acetate (PMA) (10 ng/ml) plus ionomycin (0.5 µM) (positive control for degranulation). Secondly, 50% of the supernatant was transferred into alternative wells in the same 96-well plate. The remaining supernatant was discarded, adding 1% Triton to obtain total cell lysates of β-hexosaminidase. Fifty microliters of P-nitrophenyl-N-acetyl-β-D-glucopyranoside (Sigma-Aldrich) were added to each well and the plate was incubated for 1 hour at 37°C. One hundred microliters of carbonate buffer were added to each well to stop the reaction. Absorbance was read at 405 nm. Percentage of beta-hexosaminidase was calculated using the following formula: [cells degranulation/(cells degranulation + total lysates)]*100.

Degranulation responses for all reagents tested showed no significant differences from those obtained from the negative control (basal degranulation). Taking these results into consideration, we did not proceed to analyze responses with MRGPRX2-silenced cells as we initially intended, since the experiment showed no evidence of direct initial MC degranulation to compare with.

## Discussion

Our results show that neither PEG nor trometamol are capable of stimulating MC cells directly in this *in vitro* model for non-IgE-mediated hypersensitivity. In contrast with our findings with other drugs (8), no degranulation was found after incubating LAD2 cells directly. Testing for MRGPRX2-mediated activation would then have involved silencing this receptor and detecting a change in degranulation responses, along with the addition of a known receptor agonist as a positive control. We did not proceed to perform this step, as previous experience with this model (8) shows that drugs involving MRGRPX2-mediated responses stimulate LAD2 cells directly in the first step, and that LAD2 cells fully express MRGPRX2, as confirmed by flow cytometry and Western Blot studies. Interestingly, the report published by our group regarding activation showed that several drugs stimulated MCs directly in the first step, but only a fraction of them triggered this receptor specifically (11). We therefore believe that other non-IgE responses other than those mediated by this receptor, are tested by the model.

Further explorations may be required to explain these results. Firstly, acquiring complete vaccine formulations for experimental use was complicated given the restrictions from our local health authorities. Since we did not include stimulation with a complete vaccine formulation, we cannot exclude that MCs could be activated directly by other excipients in the vaccine. Furthermore, Troelnikov et al. showed that, in PEG-allergic patients, PEG in its native form was unable to induce basophil degranulation while the PEGylated liposomal nanoparticles used in the Pfizer vaccine could induce cell degranulation (13). According to their report, skin test positivity may be dependent on PEG conformation or arrangement in the drug's surface (13). Thus, cell recognition of excipients may be influenced in some cases by component presentation.

On the other hand, a recent editorial by Cabanillas et al., comments on the fact that the exact molecular compounds present in SARS-CoV2 vaccines are PEG-lipid conjugates or PEGylated lyposomes (14). The authors reference a study by Warren and colleagues, which shows that patients with anaphylactic reactions to the mRNA vaccine against SARS-CoV2 had positive basophil activation test responses to a PEG-lipid conjugate, DMG-PEG 2000, the compound contained in the mRNA-1273 vaccine (15). The lack of response seen in our studies may be explained by the fact that a conjugated form of the excipients is involved in the induction of hypersensitivity, and not the excipients on their own. We believe our assays may be expanded to test whether PEG-lipid conjugates and complete vaccine formulations induce activation using this and other models.

It should be considered, however, that the research published by Warren et al., employs a model for the study of IgE-mediated activation, and that, in this report, we were specifically seeking alternative mechanisms, including activation *via* MRGPRX2. Therefore, our model does not explore IgE-mediated degranulation. Our findings do not contradict the recent reports of anti-PEG IgE found in the context of anaphylaxis to PEG (10). Our model does not necessarily test for complement-related activation, either. A mechanism of complement-mediated activation dependent on anti-PEG immunoglobulin M (IgM), for example, would not be detected. Further experiments exploring IgE, IgM, or immunoglobulin G mechanisms of hypersensitivity are warranted to further clarify the pathways involved in these reactions and predict their clinical behavior.

Furthermore, conformational considerations may apply to elements other than excipients tested. As far as we know, for example, MRGPRX2 expression in the LAD2 MC line is restricted to a single genetic variant. Studies using variants of the receptor gene in transfected LAD2 cells may clarify whether excipients induce direct activation in cells expressing different versions of the receptor.

Finally, even though cell viability does not seem to be an issue at the concentrations employed in our study, general limitations of *in vitro* models should be considered.

In conclusion, in an *in vitro* model for the study of non-IgE-mediated mechanisms of activation by drugs, which has previously shown to be useful in the study of reactions mediated by MRGPRX2, PEG and trometamol, in their pure form, did not induce MC activation. This result is different from results published with this previously validated model (11), in which several drugs caused MC degranulation after incubation, and where only morphine, vancomycin and cisatracurium specifically triggered the receptor, as assessed by changes in beta-hexosaminidase release (11). If these components in pure state are in fact responsible for the observed cases of immediate-type hypersensitivity direct activation of MCs as evaluated by this model, does not seem to be involved. Studies using complete vaccine formulations, PEGylated liposomes or PEG/lipid conjugates, and receptor gene variants, are needed to further consolidate mechanisms of vaccine hypersensitivity.

## Data Availability

The original contributions presented in the study are included in the article, further inquiries can be directed to the corresponding author.
